# Phylogeography and palaeodistribution modelling of *Nassauvia* subgenus *Strongyloma* (Asteraceae): exploring phylogeographical scenarios in the Patagonian steppe

**DOI:** 10.1002/ece3.1268

**Published:** 2014-10-24

**Authors:** Marcela V Nicola, Silvana M Sede, Raúl Pozner, Leigh A Johnson

**Affiliations:** 1 Instituto de Botánica DarwinionC.C. 22, B1642HYD, San Isidro, Provincia de Buenos Aires, Argentina; 2 Department of Biology and Bean Life Science Museum, Brigham Young University4102 LSB, Provo, Utah, 84602, USA

**Keywords:** Colonization, fragmentation, glacial refugia, Patagonian steppe, plant phylogeography, Pleistocene glaciations, range expansion, species distribution modelling

## Abstract

The Patagonian steppe is an immense, cold, arid region, yet phylogeographically understudied. *Nassauvia* subgen. *Strongyloma* is a characteristic element of the steppe, exhibiting a continuum of morphological variation. This taxon provides a relevant phylogeographical model not only to understand how past environmental changes shaped the genetic structure of its populations, but also to explore phylogeographical scenarios at the large geographical scale of the Patagonian steppe. Here, we (1) assess demographic processes and historical events that shaped current geographic patterns of haplotypic diversity; (2) analyze hypotheses of isolation in refugia, fragmentation of populations, and/or colonization of available areas during Pleistocene glaciations; and (3) model extant and palaeoclimatic distributions to support inferred phylogeographical patterns. Chloroplast intergenic spacers, *rpl32–trnL* and *trnQ–5′rps16*, were sequenced for 372 individuals from 63 populations. Nested clade analysis, analyses of molecular variance, and neutrality tests were performed to assess genetic structure and range expansion. The present potential distribution was modelled and projected onto a last glacial maximum (LGM) model. Of 41 haplotypes observed, ten were shared among populations associated with different morphological variants. Populations with highest haplotype diversity and private haplotypes were found in central-western and south-eastern Patagonia, consistent with long-term persistence in refugia during Pleistocene. Palaeomodelling suggested a shift toward the palaeoseashore during LGM; new available areas over the exposed Atlantic submarine platform were colonized during glaciations with postglacial retraction of populations. A scenario of fragmentation and posterior range expansion may explain the observed patterns in the center of the steppe, which is supported by palaeomodelling. Northern Patagonian populations were isolated from southern populations by the Chubut and the Deseado river basins during glaciations. Pleistocene glaciations indirectly impacted the distribution, demography, and diversification of subgen. *Strongyloma* through decreased winter temperatures and water availability in different areas of its range.

## Introduction

Phylogeographical reconstructions along large, extensive geographic areas are difficult to assess because they ideally need widely distributed, ubiquitous taxa, present at regional scale as a characteristic element of different local communities. *Nassauvia* Comm. ex Juss. subgen. *Strongyloma* (DC) Cabrera (Asteraceae) is one of these rare cases; it is a typical element throughout the immense territory of the Patagonian steppe.

Cabrera (1982) distinguished five species within subgen. *Strongyloma* by *α* taxonomy: *Nassauvia axillaris* (Lag. ex Lindl.) D. Don, *Nassauvia fuegiana* (Speg.) Cabrera, *Nassauvia glomerulosa* (Lag. ex Lindl.) D. Don, *Nassauvia maeviae* Cabrera, and *Nassauvia ulicina* (Hook. f.) Macloskie. The subgenus as a whole is easy to recognize in the field: it comprises small shrubs with a tendency to heteroblasty, with 1–5 white flowers per capitulum, and hairy cypselas (Cabrera 1982; Freire et al. 1993). Although Cabrera (1982) suggested wind dispersal, our personal observations in the field suggest that cypselas are dispersed mainly rolling on the ground, because the pappus is quickly deciduous. Distinguishing species within this complex is challenging; however, because variation in morphological traits does not strictly follow species boundaries as traditionally defined (Nicola et al. 2014). Maraner et al. (2012) found that subgen. *Strongyloma* is polyphyletic based on the ITS region of nuclear ribosomal DNA, but this appears to be an error in sample or sequence identity, or the use of divergent sequence paralogs (Nicola et al. 2014; Nicola, M. V., S. M. Sede, L. A. Johnson, and R. Pozne in preparation). Morphological evidence (Cabrera 1982; Freire et al. 1993; Nicola et al. 2014) and molecular data derived from plastid and nuclear ribosomal DNA regions (Nicola, M. V., S. M. Sede, L. A. Johnson, and R. Pozner in preparation) indicate subgen. *Strongyloma* is a well-supported monophyletic group with wide and continuous morphological variation coupled with little genetic divergence. We follow here the taxonomical decision of Nicola, M. V., S. M. Sede, L. A. Johnson, and R. Pozner (in preparation) by considering *Nassauvia* subgen. *Strongyloma* as a monospecific taxon. The morphological combinations traditionally accepted as species (Cabrera 1982; Freire et al. 1993) are here referred as “morphological variants” (i.e., axillaris, glomerulosa, fuegiana, maeviae, and ulicina morphologies), just to allow some reference points within the morphological, continuous variation observed in this group of plants (Nicola et al. 2014).

*Nassauvia* subgen. *Strongyloma* has a remarkably wide distribution, mainly in the Patagonian steppe with a few enclaves in the Andean region, from sea level to 4560 m a.s.l., in southern Bolivia (*c*. 21°S), in Argentina (from Jujuy Province *c*. 22°S to northern Tierra del Fuego Province *c*. 54°S), and in Chile (from Valparaíso *c*. 32°S to Magallanes *c*. 51°S), including a variety of climatic zones but mainly adapted to extremely xeric environments (Cabrera 1982; Nicola et al. 2014). With a particularly abundant distribution, populations with axillaris and glomerulosa morphological variants characterized the Western and the Central Patagonian districts of the Patagonian Phytogeographical Province, respectively (Cabrera and Willink 1980). Sympatric assemblages of different combinations of two or three morphological variants of subgen. *Strongyloma* are frequent in some areas of Patagonia. With abundant morphologically intermediate individuals, such areas might represent secondary contact zones or populations with incomplete sorting of lineages (Nicola et al. 2014). The fossil record refers the oldest members of subtribe Nassauviinae back to the Miocene, when progenitors of this group were expanding in eastern Patagonia (Barreda et al. 2008). Nassauviinae, together with Mutisieae, are among the oldest, early branching groups within Asteraceae, after Onoseridae and Barnadesioideae (Funk et al. 2009).

The vast, flat, arid Patagonian steppe ecoregion is longitudinally placed from the east of the slopes of the southern Andes to the Atlantic coast, and latitudinally located from central Mendoza Province (*c*. 32°S) to northern Tierra del Fuego Province (*c*. 54°S) in Argentina, covering a total area of approximately 787,000 km^2^ (Correa 1998). It was shaped during the Middle–Late Miocene (16–5.3 Ma) mainly by the uplift of the Andes. This orogeny blocked and filtered moist winds coming from the Pacific Ocean and produced a substantial rain shadow to the east, resulting in cold and dry conditions throughout the steppe, a contraction of the forest, and the expansion of xerophytic taxa (Barreda and Palazzesi 2007). Major glacial–interglacial cycles in Patagonia began during the Late Miocene (*c*. 5–6 Ma), with ice reaching its maximum development in the Early Pleistocene (*c*. 1.15 Ma; Ogg et al. 2008; Rabassa et al. 2011). The major glacial advances in southern South America were the Great Patagonian Glaciation (GPG; *c*. 1 Ma) and the Last Glacial Maximum (LGM; *c*. 21 Ka). The GPG resulted in the greatest expansion of ice sheets in the Patagonian steppe compared with LGM and other Pleistocene glaciations (Rabassa et al. 2011). However, a large part of the steppe remained free of ice during the glacial episodes, and climatic conditions were less severe than those occurring in North America (Markgraf et al. 1995).

In Patagonia, Pleistocene glaciations caused several climatic and environmental changes. The sea level lowered, shifting the coastline about four degrees eastward. Climatic continentality of the surrounding areas enhanced, with rising extreme temperatures and decreased precipitation. The Argentinean submarine platform was partially exposed, substantially increasing the space available for animal and plant colonization (Rabassa 2008). Although these climatic and environmental changes are thought to have affected species distribution, the Patagonian steppe remains poorly phylogeographically studied (Sérsic et al. 2011). Plant studies have focused mainly on the Andean region (e.g., Muellner et al. 2005; Marchelli and Gallo 2006; Amico and Nickrent 2009; Acosta and Premoli 2010; Vidal-Russell et al. 2011; Segovia et al. 2012; among others), although interest in understanding the evolutionary history and processes of plant diversification in the Patagonian steppe has increased in recent years (Jakob et al. 2009; Cosacov et al. 2010, 2013; Sede et al. 2012). Considering this background, subgen. *Strongyloma* is a relevant plant phylogeographical model not only to understand how recent past environmental changes shaped the genetic structure of its populations, but also to explore regional phylogeographical scenarios. Here, we examine the relative importance of probable historical events and/or past demographic processes that can explain the current genetic population structure in subgen. *Strongyloma* through phylogeographical analyses using plastid DNA sequences. We also investigate how palaeoclimatic conditions have influenced the distribution of this subgenus using distribution modelling (Hijmans and Graham 2006). We analyze three hypotheses: (1) the existence of multiple glacial refugia in flanking zones of the Andes covered by ice followed by postglacial expansion; (2) the expansion of populations over the current marine platform during the Pleistocene and their successive, recent retraction to the surroundings of the current Atlantic coast of Patagonia; and/or (3) the fragmentation of populations that persisted in areas not reached by the ice sheet during glacial periods in the center of the steppe due to past river basins dynamic. Under the first scenario, population expansion from refugia should leave genetic signatures of high diversity and private haplotypes in refugial areas, produced by considerable genome divergence and reorganization. From the perspective of the second hypothesis, in recently reduced areas nearby the Atlantic coast with rapid population retraction, diversity should be low due to successive bottlenecks on the colonizing genomes that lead to a loss of alleles. Lastly, if populations persisted in more or less fragmented areas, then genetic diversity and unique haplotypes within populations should be low: during posterior expansions, haplotypes may spread slowly and more equally (Hewitt 1996). Finally, we search for significant phylogeographical breaks consistent with those found previously for other Patagonian organisms and consider these breaks with prior hypotheses (Sérsic et al. 2011; and literature therein; Sede et al. 2012; Cosacov et al. 2013).

## Materials and Methods

### Sampling

**Figure 1:**
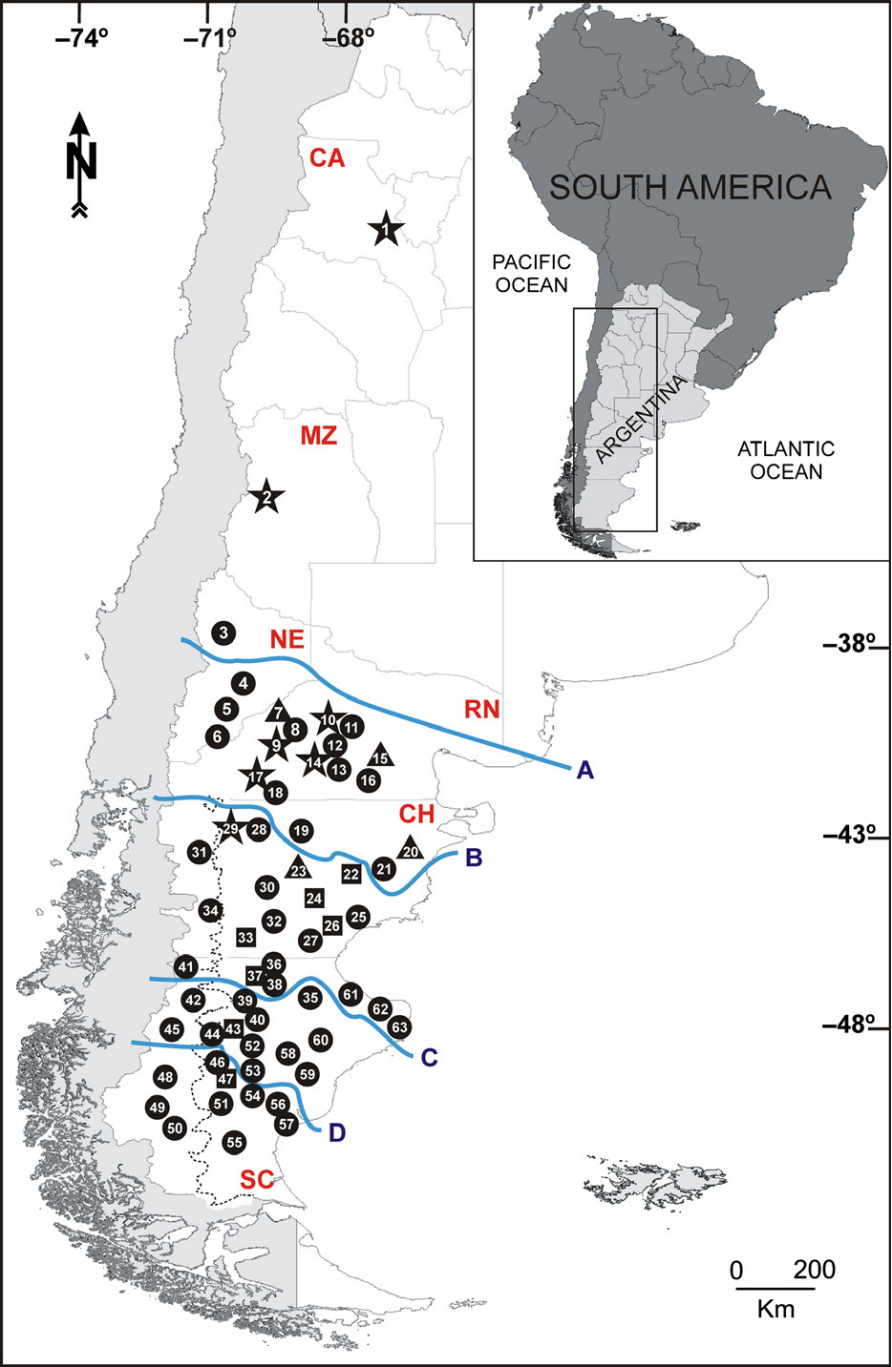
The inset depicts a geographic map of South America with the study area indicated in a box. The main map shows the locations of the 63 sampled populations of *Nassauvia* subgen. *Strongyloma*. Locality numbers correspond to those in Table 1. Symbols indicate four of the five morphological variants: stars, axillaris; triangles, fuegiana; circles, glomerulosa; and squares, ulicina. Acronyms designate the Argentinean provinces sampled: CA, Catamarca; CH, Chubut; MZ, Mendoza; NE, Neuquén; RN, Río Negro; and SC, Santa Cruz. The solid lines illustrate schematically the main rivers considered in the analyses: A. Agrio, Neuquén, and Negro rivers; B. Chubut River; C. Deseado River; and D. Chico River. The dotted line shows the limit of the ice during the Great Patagonian Glaciation (GPG; Rabassa et al. 2011).

A total of 372 individuals from 63 populations were sampled in Argentina (excepting Tierra del Fuego), covering most of the distribution of subgen. *Strongyloma* and its wide morphological variation, including axillaris, glomerulosa, fuegiana, and ulicina morphological variants (Table 1, Fig. 1). The maeviae morphological variant is known only from the type collection, and DNA could not be obtained from the herbarium voucher. Fresh leaves were sampled from one to eight individuals at each site, separated by at least 10 m along a linear transect to minimize the potential of sibling relationships among samples. Herbarium vouchers were identified following Cabrera (1982) and deposited at SI (Instituto de Botánica Darwinion) or CORD (Museo Botánico, Universidad Nacional de Córdoba).

**Table 1:** Sampling sites (*N*_loc_), collectors, and collection numbers (herbaria in parenthesis), morphological variant, collection localities, elevation (meters), geographic coordinates, and sample size (*N*_ind_) of the *Nassauvia* subgen. *Strongyloma* populations studied in Argentina. Localities are numbered consecutively, as shown on the map in Figure 1

*N*_loc_	Legit (herbarium)	Morphology	Province	Sample location	Elevation	Latitude (°S)	Longitude (°W)	*N*_ind_
1	Zuloaga 11934 (SI)	axillaris	Catamarca	Minas Capillitas	3740	27°21′	66°20′	7
2	Zavala 57 (SI)	axillaris	Mendoza	Laguna del Diamante	2622	34°14′	69°25′	8
3	Nicola 127 (SI)	glomerulosa	Neuquén	El Huecú	1483	37°42′	70°30′	7
4	Sérsic 4126 (CORD)	glomerulosa	Neuquén	Zapala	1091	39°01′	70°01′	7
5	Nicola 121 (SI)	glomerulosa	Neuquén	Catan Lil	980	39°44′	70°29′	5
6	Sérsic 4122 (CORD)	glomerulosa	Neuquén	Collón Curá	604	40°25′	70°39′	8
7	Nicola 98 (SI)	fuegiana	Río Negro	Aguada del Trapo	775	39°56′	69°03′	2
8	Zanotti 42 (SI)	glomerulosa	Río Negro	Chasicó	1117	40°17′	68°55′	3
9	Zanotti 45 (SI)	axillaris	Río Negro	Colán Conué	1145	40°44′	69°09′	8
10	Nicola 109 (SI)	axillaris	Río Negro	Sierra de Queupuniyeu	1156	40°32′	68°11′	8
11	Nicola 108 (SI)	glomerulosa	Río Negro	Sierra Colorada	749	40°34′	67°52′	8
12	Zanotti 35 (SI)	glomerulosa	Río Negro	Los Menucos	874	40°46′	68°11′	8
13	Nicola 103 (SI)	glomerulosa	Río Negro	Prahuaniyeu	857	41°05′	67°53′	8
14	Ávila s.n.1 (SI)	axillaris	Río Negro	Aguada de Guerra	896	41°04′	68°23′	6
15	Zanotti 23 (SI)	fuegiana	Río Negro	Meseta de Somuncurá	618	40°58′	66°40′	2
16	Zanotti 24 (SI)	glomerulosa	Río Negro	Cerro Corona	1425	41°24′	66°57′	8
17	Zanotti 49 (SI)	axillaris	Río Negro	Ingeniero Jacobacci	865	41°20′	69°28′	5
18	Zanotti 53 (SI)	glomerulosa	Río Negro	Moligüe	1162	41°45′	69°22′	4
19	Nicola 5 (SI)	glomerulosa	Chubut	El Escorial	1025	42°56′	68°31′	5
20	Biganzoli 1975 (SI)	fuegiana	Chubut	Dolavon	95	43°21′	65°56′	1
21	Nicola 62 (SI)	glomerulosa	Chubut	Dique Florentino Ameghino	260	43°41′	66°29′	7
22	Nicola 66 (SI)	ulicina	Chubut	Las Plumas	270	43°56′	67°18′	2
23	Nicola 76 (SI)	fuegiana	Chubut	Los Altares	250	43°50′	68°35′	2
24	Nicola 75 (SI)	ulicina	Chubut	Meseta del Canquel	364	44°30′	68°12′	2
25	Paiaro s.n.43 (SI)	glomerulosa	Chubut	Pampa Salamanca	579	45°08′	67°09′	6
26	Nicola 74 (SI)	ulicina	Chubut	Puente Nollman	269	45°18′	67°46′	2
27	Nicola 58 (SI)	glomerulosa	Chubut	Pampa del Castillo	364	45°33′	68°11′	4
28	Nicola 8 (SI)	glomerulosa	Chubut	Paso del Sapo	584	42°45′	69°43′	8
29	Nicola 14 (SI)	axillaris	Chubut	Piedra Parada	540	42°41′	70°09′	1
30	Nicola 33 (SI)	glomerulosa	Chubut	Cañadón Grande	900	44°15′	69°24′	7
31	Nicola 17 (SI)	glomerulosa	Chubut	Tecka	676	43°15′	70°52′	8
32	Nicola 29 (SI)	glomerulosa	Chubut	Matasiete	854	45°07′	69°20′	8
33	Nicola 170 (SI)	ulicina	Chubut	Río Mayo	421	45°28′	69°50′	1
34	Nicola 28 (SI)	glomerulosa	Chubut	Alto Río Senguer	643	44°48′	70°42′	8
35	Nicola 55 (SI)	glomerulosa	Santa Cruz	Pico Truncado	268	46°58′	68°25′	7
36	Biganzoli 2345 (SI)	glomerulosa	Santa Cruz	Las Heras 1	343	46°36′	69°27′	6
37	Biganzoli 2347 (SI)	ulicina	Santa Cruz	Las Heras 2	343	46°37′	69°28′	8
38	Paiaro s.n.42 (SI)	glomerulosa	Santa Cruz	Las Heras 3	336	46°36′	69°27′	7
39	Paiaro s.n.40 (SI)	glomerulosa	Santa Cruz	Estancia Sol de Mayo	822	47°22′	69°49′	8
40	Paiaro s.n.38 (SI)	glomerulosa	Santa Cruz	Estancia Piedra Grande	775	47°36′	69°46′	8
41	Paiaro s.n.16 (SI)	glomerulosa	Santa Cruz	Meseta del Guenguel	739	46°12′	71°35′	8
42	Paiaro s.n.12 (SI)	glomerulosa	Santa Cruz	Meseta Lago Buenos Aires	1353	46°58′	71°06′	8
43	Nicola 167 (SI)	ulicina	Santa Cruz	Estancia La Bajada	473	47°58′	70°24′	1
44	Zanotti 117 (SI)	glomerulosa	Santa Cruz	Estancia El Delfín	618	47°58′	70°58′	8
45	Paiaro s.n.34 (SI)	glomerulosa	Santa Cruz	Estancia Cerro Beltza	912	48°00′	71°41′	8
46	Paiaro s.n.19 (SI)	glomerulosa	Santa Cruz	Gobernador Gregores 1	459	48°51′	70°36′	6
47	Nicola 157 (SI)	ulicina	Santa Cruz	Gobernador Gregores 2	434	48°50′	70°33′	1
48	Zanotti 98 (SI)	glomerulosa	Santa Cruz	Meseta de San Adolfo	597	49°11′	71°53′	8
49	Zanotti 93 (SI)	glomerulosa	Santa Cruz	La Leona	294	49°51′	72°02′	8
50	Zanotti 89 (SI)	glomerulosa	Santa Cruz	Río Bote	234	50°18′	71°39′	8
51	Paiaro s.n.27 (SI)	glomerulosa	Santa Cruz	Cerro Mank Aike	807	49°46′	70°44′	3
52	Paiaro s.n.36 (SI)	glomerulosa	Santa Cruz	Estancia Dos Manantiales	741	48°15′	69°47′	6
53	Zanotti 66 (SI)	glomerulosa	Santa Cruz	Estancia 8 hermanos	331	48°48′	69°45′	8
54	Zanotti 69 (SI)	glomerulosa	Santa Cruz	Estancia La Delfina	121	49°28′	69°41′	6
55	Paiaro s.n.24 (SI)	glomerulosa	Santa Cruz	Estancia El Mendocino	332	50°42′	70°14′	4
56	Zanotti 70 (SI)	glomerulosa	Santa Cruz	Comandante Luis Piedra Buena	108	49°54′	69°00′	8
57	Zanotti 71 (SI)	glomerulosa	Santa Cruz	Puerto Santa Cruz	126	50°03′	68°53′	8
58	Nicola 47 (SI)	glomerulosa	Santa Cruz	Estancia Vega Grande	214	48°30′	68°52′	7
59	Zanotti 64 (SI)	glomerulosa	Santa Cruz	Estancia Cerro Perdido	241	48°58′	68°29′	7
60	Nicola 51 (SI)	glomerulosa	Santa Cruz	Estancia Piedra Negra	221	48°09′	68°13′	8
61	Biganzoli 2343 (SI)	glomerulosa	Santa Cruz	Fitz Roy	242	46°58′	67°16′	3
62	Nicola 37 (SI)	glomerulosa	Santa Cruz	Estancia El Polvorín	106	47°07′	66°28′	7
63	Zanotti 142 (SI)	glomerulosa	Santa Cruz	Puerto Deseado	16	47°43′	65°50′	5

### DNA isolation, amplification, and sequencing

Genomic DNA was isolated from silica dried leaf tissue following a modified cetyl trimethyl ammonium bromide (CTAB) protocol (Doyle and Doyle 1987; Cullings 1992). The chloroplast intergenic spacers *rpl32–trnL* (primers rpL32-F and trnL^UAG^; Shaw et al. 2007) and *trnQ–5′rps16* (primers trnQ^UUG^ and rps^16 × 1^; Shaw et al. 2007) were selected as markers because they showed the highest sequence variability among several surveyed regions.

DNA was amplified and sequenced with a profile consisting of 94°C for 3 min followed by 30 cycles of 94°C for 1 min, 50 or 52°C for 1 min for *trnQ–5′rps16* and *rpl32–trnL,* respectively, and 72°C for 1 min. Amplification products were purified using PCR_96_ cleanup plates (Millipore Corp, Billerica, MA), sequenced with BigDye 3 (Applied Biosystems, Foster City, CA), and purified with Sephadex (GE Healthcare, Piscataway, NJ) before electrophoresis on an AB 3730xl automated sequencer housed in Brigham Young University's DNA Sequencing Centre. Electropherograms were edited and assembled using Sequencher 4.6 (Gene Codes, Ann Arbor, MI). Sequences were aligned with ClustalX 1.81 (Thompson et al. 1997) with subsequent manual adjustments using BioEdit 5.0.9 (Hall 1999). Indels were coded as binary characters using simple indel coding (Simmons and Ochoterena 2000) as implemented in SeqState 1.4 (Müller 2005). The two chloroplast regions were concatenated into a single matrix a priori given their shared ancestry, maternal inheritance, and extremely rare instances of recombination within the chloroplast genome. Unique sequences from this combined matrix were identified as haplotypes. All sequences were deposited in GenBank (*rpl32–trnL* KM978451–KM978491 and *trnQ–5′rps16* KM978492–KM978532).

### Haplotype network and geographic distribution

Nested clade analysis (NCA) was performed following Templeton et al. (1995) and Templeton (2004) using ANeCA 1.2 (Panchal 2007). Although NCA has been criticized (Petit 2007; Beaumont and Panchal 2008; Knowles 2008), it has been validated extensively (Templeton 2008, 2009) and is used as an exploratory analysis in conjunction with other population genetic parameters to draw inferences here.

A haplotype network was constructed using TCS 1.21 (Clement et al. 2000) with the default 0.95 probability connection limit. Ambiguous connections (loops) were resolved using predictions from coalescent theory and information about sampling according to three criteria: frequency, network topology, and geography (Crandall and Templeton 1993).

The resolved haplotype network was converted into a hierarchical nested design following Templeton et al. (1987) and Templeton and Sing (1993). Clade (Dc) and nested clades (Dn) distances were estimated to assess association between the nested cladogram and geographic distances among sampled localities (Templeton et al. 1995) using GeoDis 2.6 (Posada et al. 2000). Null distributions (i.e., under a hypothesis of no geographic association of clades and nested clades) for permutational contingency table test comparisons were generated from 10,000 Monte Carlo replications, with a 95% confidence level. For significant associations, the inference key of Templeton (2011) was used to recognize probable demographic processes and/or historical events of the clades.

### Population genetic analyses

Haplotype diversity (*h*; Nei 1987), nucleotide diversity (*π*; mean number of pairwise differences per site; Nei 1987), and mean number of pairwise differences (*p*; Tajima 1983) were calculated for the subgenus as a whole, each sampling location, and each geographic region, using DnaSP 5.10.01 (Librado and Rozas 2009).

To investigate hierarchical levels of population structure, two analyses of molecular variance (AMOVA) were performed that consider genetic distances between haplotypes and their frequencies using Arlequin 3.5.1.2 (Excoffier and Lischer 2010). First, populations were grouped according to different latitudinal regions, defined by major rivers: (1) north of Agrio, Neuquén, and Negro rivers, *c*. 38°S; (2) between Agrio, Neuquén, and Negro rivers, and Chubut River, *c*. 38°–43°S; (3) between Chubut and Deseado rivers, *c*. 43°–47°S; (4) between Deseado and Chico rivers, *c*. 47°–50°S; and (5) south of Chico River, *c*. 50°S (Fig. 1). The aim of the second analysis was to test whether genetic variation was significantly structured among glaciated and nonglaciated sites during GPG in populations south of Chubut River (Rabassa et al. 2011), following a longitudinal transect at *c*. 71°W. This region was selected given that eight of the 63 populations were located within the ice zone (Fig. 1). Estimation of molecular diversity indexes and AMOVAs were performed considering only populations with four or more sampled individuals.

### Demographic history analyses

To analyze the demographic history of populations in each geographic area, Tajima's *D* (Tajima 1989), Fu's *F*_S_ (Fu 1997), and Ramos-Onsins and Rozas' *R*_2_ (Ramos-Onsins and Rozas 2002) statistics of neutrality were calculated to test for evidence of range expansion. Significant negative values of *D* and *F*_S_ and small positive values of *R*_2_ indicate an excess of low frequency mutations relative to expectations under the standard neutral model (i.e., strict selective neutrality of variants, constant population size, and lack of subdivision and gene flow). *F*_S_ and *R*_2_ are the most powerful tests used to detect population growth (Ramos-Onsins and Rozas 2002). Significance was evaluated by comparing observed values with null distributions generated by 10,000 replicates, using the empirical population sample size and observed number of segregating sites implemented by DnaSP 5.10.01.

### Distribution modelling

To validate the phylogeographical analyses, we modelled the present potential geographic distribution of *Nassauvia* subgen. *Strongyloma* and projected it onto a LGM model about 21 Ka using point locality information and environmental data in Diva-Gis 7.3 (Hijmans et al. 2001) and MaxEnt 3.3.3, with the maximum entropy machine-learning algorithm (Phillips et al. 2004).

We recorded latitudinal and longitudinal coordinates from 224 localities of subgen. *Strongyloma*, covering its entire distribution range primarily using a handheld geographical positioning system (GPS) unit. Only 36 point localities of the total were geo-referenced using Google Earth (http://www.google.com/earth/index.html). Environmental data with a resolution of 2.5 arc minutes (5 km^2^) for current and past conditions were downloaded from the WorldClim database 1.4 (Hijmans et al. 2005) and were represented by 19 bioclimatic variables derived from the monthly temperature and rainfall values.

Current conditions are interpolations of observed data from climate stations around the world, representing a 50-year period from 1950 to 2000. Past conditions for the LGM are calibrated and statistically downscaled reconstructions based on the WorldClim data for current conditions. We chose the prediction of one of two global climate models tested for the LGM, the community climate system model (CCSM) and the Model for Interdisciplinary Research on Climate (MIROC), by evaluating the MaxEnt values of the area under the curve (AUC) and interpreting the resulting maps of each model in Diva-Gis.

We generated 2000 random points from across Argentina, Bolivia, and Chile using Geo Midpoint (http://www.geomidpoint.com/random/) and extracted environmental data in Diva-Gis. To avoid overestimation of climatic data that can lead to misleading results (Phillips et al. 2004; Peterson and Nakazawa 2008), we calculated Pearson's correlation coefficients (*r* ≥ 0.75) and performed Principal Component Analyses in InfoStat 2.0 (Di Rienzo et al. 2011) following a similar procedure described by Werneck et al. (2011) and Sede et al. (2012). We identified highly correlated variables and selected ten that we considered more biologically meaningful and directly relevant to *subgen. Strongyloma*. These were the annual mean temperature (Bio 1), isothermality (Bio 3), temperature annual range (Bio 7), mean temperature of the wettest quarter (Bio 8), mean temperature of the driest quarter (Bio 9), mean temperature of the coldest quarter (Bio 11), annual precipitation (Bio 12), precipitation seasonality (Bio 15), precipitation of the warmest quarter (Bio 18), and precipitation of the coldest quarter (Bio 19).

The bioclimatic layers were trimmed to the surrounding areas of the known geographic distribution of subgen. *Strongyloma*, and then projected over a wider region from latitude 17°41′S to 55°49′S and from longitude 57°31′W to 75°43′O for the present and the LGM in Diva-Gis.

MaxEnt was run using the following settings for current and past models: 10 replicates with auto features, response curves, jackknife tests, logistic output format, random seed, random test percentage = 25% (the test data are a random sample taken from the 100% of the species presence localities, which allows the program to do some simple statistical analysis; therefore, the remaining 75% represents the training data), replicate run type = cross-validate, regularization multiplier = 1, maximum iterations = 500, convergence threshold = 0.00001, and maximum number of background points = 10,000. To determine the threshold value for each prediction, we used the value of the minimum training presence logistic threshold. Variable importance was determined comparing percent contribution values and jackknife plots.

## Results

### DNA data sets

DNA sequences of subgen. *Strongyloma* ranged from 913 to 928 base pairs (bp) for *rpl32–trnL* (with two indels of seven and eight bp) and 916 to 925 bp for *trnQ–5′rps16* (with one indel of nine bp). The three indels were coded as present or absent and appended to the end of the matrix. The combined matrix included 1856 characters, of which 37 were variable.

### Haplotype network and geographic distribution of haplotypes

**Figure 2:**
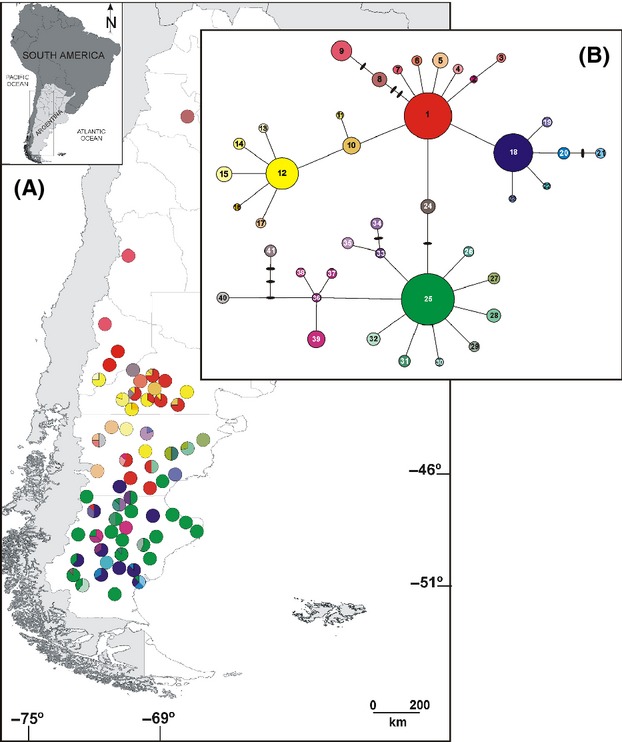
Geographic distribution and genealogical relationships of the chloroplast DNA haplotypes found in *Nassauvia* subgen. *Strongyloma*. (A) Pie charts reflect the frequency of occurrence of each haplotype in each population. Haplotype colors correspond to those shown in panel (B). (B) Statistical parsimony network linking the 41 haplotypes. Sampled haplotypes are designated by numbers, and circle sizes are proportional to haplotype frequencies. The solid bars represent hypothetical haplotypes not observed in the analyzed individuals.

Statistical parsimony retrieved a well-resolved network in which three closed loops were resolved unambiguously (Fig. 2). A total of 41 haplotypes (H) were identified. Eleven haplotypes occurred in interior positions and 30 at tip positions. Nine additional haplotypes were not observed in the analyzed individuals and occur only as inferred intermediates in the network. Four frequent, widespread haplotypes were found (H25, H1, H18, and H12; Table 2, Fig. 2).

**Table 2:** Distribution of haplotypes of *Nassauvia* subgen. *Strongyloma* between individuals, populations, morphological variants, Argentinean provinces, and haploclades

Haplotype	*N*_ind_	%_ind_	*N*_pop_	%_pop_	Morphology	Distribution	Geographic region
1	60	0.161	13	0.127	AXI-ULI	NE-RN-CH-SC	NP1
2	1	0.003	1	0.010	GLO	CH	NP1
3	2	0.005	1	0.010	GLO	CH	NP1
4	2	0.005	1	0.010	GLO	CH	NP1
5	8	0.022	1	0.010	GLO	CH	NP1
6	3	0.008	1	0.010	GLO	RN	NP1
7	2	0.005	1	0.010	GLO	SC	NP1
8	7	0.019	1	0.010	AXI	CA	HA
9	15	0.040	2	0.020	AXI-GLO	MZ-NE	HA
10	11	0.030	3	0.025	AXI-GLO	RN	NP2
11	1	0.003	1	0.010	AXI	RN	NP2
12	20	0.054	8	0.067	AXI-FUE-GLO	NE-RN-CH	NP2
13	2	0.005	1	0.010	GLO	NE	NP2
14	4	0.011	1	0.010	AXI	RN	NP2
15	8	0.022	1	0.010	GLO	CH	NP2
16	1	0.003	1	0.010	GLO	RN	NP2
17	3	0.008	2	0.020	AXI-GLO	CH	NP2
18	38	0.102	9	0.075	GLO-ULI	CH-SC	SP2
19	3	0.008	1	0.010	GLO	SC	SP2
20	4	0.011	3	0.025	GLO	SC	SP2
21	3	0.008	1	0.010	GLO	SC	SP2
22	1	0.003	1	0.010	ULI	SC	SP2
23	1	0.003	1	0.010	GLO	SC	SP2
24	7	0.019	2	0.020	GLO	CH	SP1
25	102	0.274	23	0.192	GLO-ULI	CH-SC	SP1
26	3	0.008	1	0.010	ULI	SC	SP1
27	4	0.011	3	0.025	FUE-GLO-ULI	CH	SP1
28	6	0.016	2	0.020	GLO-ULI	CH	SP1
29	3	0.008	1	0.010	GLO	SC	SP1
30	1	0.003	1	0.010	GLO	SC	SP1
31	4	0.011	1	0.010	GLO	SC	SP1
32	5	0.013	1	0.010	GLO	SC	SP1
33	3	0.008	1	0.010	GLO	SC	CP2
34	4	0.011	1	0.010	ULI	SC	CP2
35	4	0.011	1	0.010	GLO	CH	CP2
36	2	0.005	1	0.010	GLO	SC	SP1
37	3	0.008	1	0.010	GLO	SC	SP1
38	2	0.005	1	0.010	GLO	NE	SP1
39	11	0.030	2	0.020	GLO	SC	SP1
40	4	0.011	1	0.010	GLO	CH	CP1
41	4	0.011	2	0.020	AXI-FUE	RN	CP1

*N*_ind_, number of individuals per haplotype; %_ind_, percentage of individuals per haplotype; *N*_pop_, number of populations per haplotype; %_pop_, percentage of populations per haplotype; morphological variants: AXI, axillaris; FUE, fuegiana; GLO, glomerulosa; and ULI, ulicina; Distribution: CA, Catamarca; CH, Chubut; MZ, Mendoza; NE, Neuquén; RN, Río Negro; and SC, Santa Cruz; Geographic region: CP1, central Patagonia 1; CP2: central Patagonia 2; HA, high-Andean; NP1, northern Patagonia 1; NP2, northern Patagonia 2; SP1, southern Patagonia 1; SP2, southern Patagonia 2.

Among the total observed haplotypes (Table 2), eight were shared by populations associated with different morphological variant pairs: H41 in axillaris–fuegiana, H9, H10, and H17 in axillaris–glomerulosa, H1 in axillaris–ulicina, and H18, H25, and H28 in glomerulosa–ulicina. Two additional haplotypes were found in populations associated with different combinations of three morphological variants: H12 in axillaris–fuegiana–glomerulosa and H27 in fuegiana–glomerulosa–ulicina.

**Figure 3:**
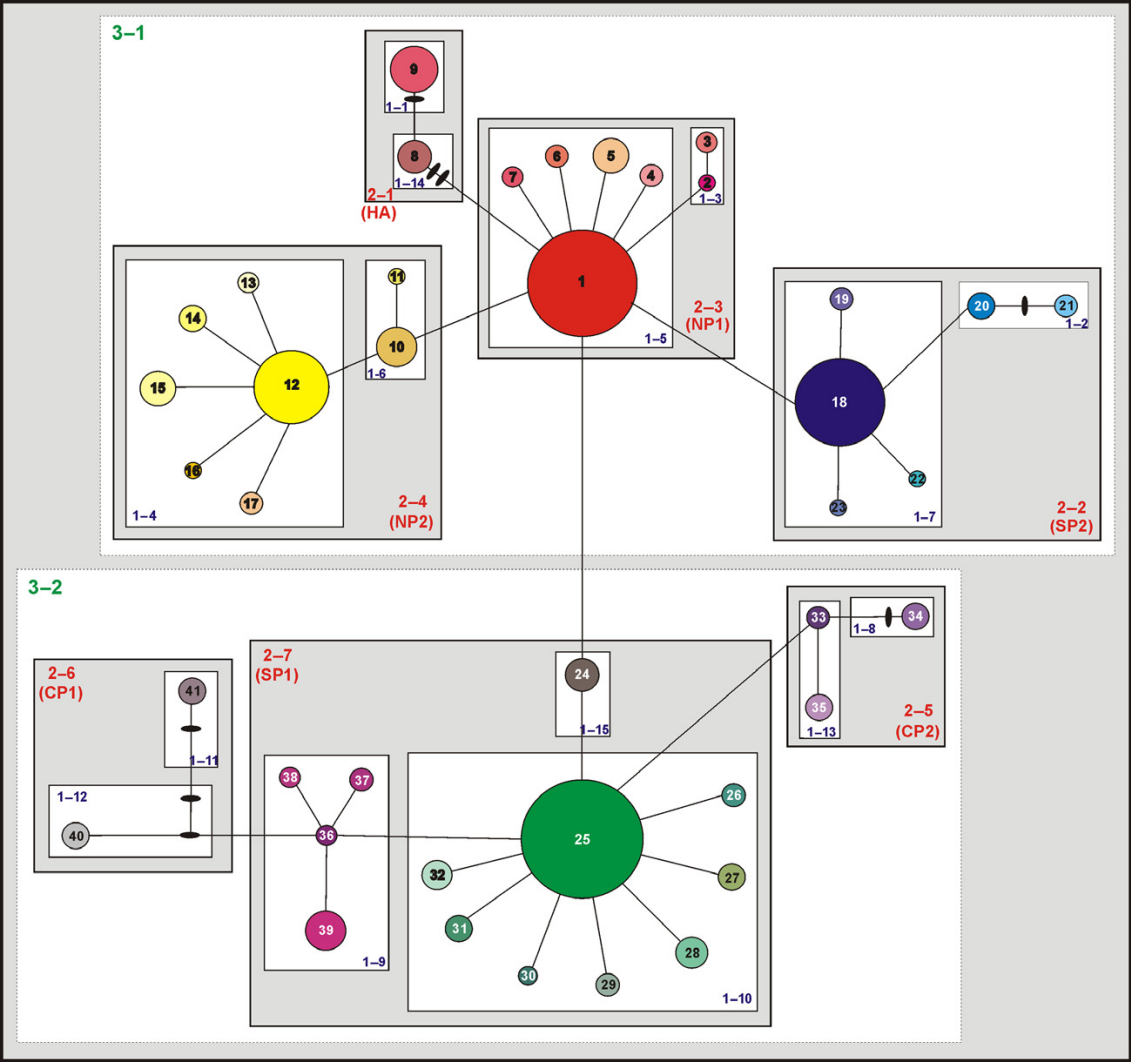
Statistical parsimony network and resulting nested clade design of the 41 haplotypes found in *Nassauvia* subgen. *Strongyloma*. Haplotypes correspond to those in Figure 2; solid bars represent hypothetical haplotypes not observed in the analyzed individuals. Haplotypes belonging to the same clade level are boxed. Acronyms associated to 2-step clades indicate the geographic region to which they belong: CP1, central Patagonia 1; CP2: central Patagonia 2; HA, high-Andean; NP1, northern Patagonia 1; NP2, northern Patagonia 2; SP1, southern Patagonia 1; SP2, southern Patagonia 2.

The nested network resulted in 15 one-step clades, seven 2-step clades, two 3-step clades, and the total cladogram (Fig. 3). Although not strictly nonoverlapping, a latitudinal distribution of the two-step clades was observed (Figs. 2, 3). Haplotypes in clade 2-1 (HA: high*–*Andean region) were distributed through the Andes north of 38°S, clearly separated from the rest. Haplotypes in clade 2-3 (NP1: northern Patagonia 1 region) included the second most frequent, widespread haplotype (H1) and were distributed between 39° and 47°S, while haplotypes in clade 2-4 (NP2: northern Patagonia 2 region) included the fourth most frequent, widespread haplotype (H12) and were distributed between 40°25′ and 44°S. Haplotypes in clades 2-6 (CP1: central Patagonia 1 region) and 2-5 (CP2: central Patagonia 2 region) were scarce and distributed between 40° and 43°15′S, and 43° and 46°37′S, respectively. Haplotypes in clade 2-7 (SP1: southern Patagonia 1 region) included the most frequent, widespread haplotype (H25) and were distributed mainly south of 43°21′S, with one exception further north at 39°44′S. Finally, haplotypes in clade 2-2 (SP2: southern Patagonia 2 region) included the third most frequent, widespread haplotype (H18) and were distributed south of 45°28′S.

All geographic regions presented populations with exclusive haplotypes (Table 2, Fig. 3). The region with the largest number of exclusive haplotypes was SP1, with eight exclusive haplotypes distributed in southern Neuquén (population 5; H38) and mainly in Santa Cruz (populations 37, 39, 44, 46, 50, 53, and 58; H26, H31, H37, H36, H32, H30, and H29 respectively). The second region with the highest number of exclusive haplotypes was NP1, with six exclusive haplotypes distributed in southern Neuquén (population 5; H7), in central Río Negro (population 8; H6), and in south-western Chubut (populations 30, 32, and 34; H2, H3, H4, and H5). The NP2 region had five exclusive haplotypes distributed in southern Neuquén (population 6; H13), central and central-southern Río Negro (populations 10, 17, and 18; H11, H14, and H16), and central-northern Chubut (population 28; H15). The SP2 region had four exclusive haplotypes distributed in Santa Cruz (populations 42, 47, 49, and 57; H19, H21, H22, and H23). The CP2 region presented three exclusive haplotypes distributed in central-northern Chubut (population 19; H35) and in central-northern Santa Cruz (populations 36 and 37; H33 and H34, respectively). The CP1 region had only one exclusive haplotype in north-western Chubut (population 31; H40). The HA region possessed only one exclusive haplotype in Catamarca (population 1; H8). Exclusive haplotypes were scattered throughout the study area, although most were located in central-western and south-eastern Santa Cruz.

### Inferences from nested clade analysis

Six one-step clades (1-4, 1-5, 1-7, 1-9, 1-10, and 1-13), all seven-two-step clades, the two-three-step clades, and the total cladogram showed significant levels of geographic association, while the geographic association hypothesis was rejected for the remaining nine-one-step clades (Table 3, Fig. 3).

**Table 3:** Population processes and/or historical events affecting genetic structure in *Nassauvia* subgen. *Strongyloma* based on nested clade analysis (NCA). Geographic region, hierarchically nested clades, results of permutation contingency tests with their associated *P* value, NCA inference chain and inferred events are shown

Geographic region	Clade	*χ*^2^ statistic	*P*-value	Inference chain	Inferred pattern/event
NP2	1-4	119.60	<0.05	1-2-3-4-9-10 NO	Geographic sampling inadequate to discriminate between fragmentation and isolation by distance.
NP1	1-5	222.50	<0.05	1-2-3-5-6-7-8 YES	Restricted gene flow/dispersal but with some long-distance dispersal over intermediate areas not occupied by the species, or past gene flow followed by extinction of intermediate populations.
SP2	1-7	102.49	<0.05	1-2-3-5-6-13-14 NO	Long-distance colonization and/or past fragmentation.
SP1	1-9	42.54	<0.05	1-2-3-5-6-7 YES	Restricted gene flow/dispersal but with some long-distance dispersal.
SP1	1-10	464.99	<0.05	1-2-3-5-6-7-8 NO	Sampling design inadequate to discriminate between isolation by distance versus long-distance dispersal.
CP2	1-13	7.00	<0.05	1-19-20 NO	Inadequate geographic sampling.
HA	2-1	22.00	<0.05	1-19-20 NO	Inadequate geographic sampling.
SP2	2-2	25.33	<0.05	1-2-3-4 NO	Restricted gene flow with isolation by distance.
NP1	2-3	54.82	<0.05	–	Null hypothesis cannot be rejected.
NP2	2-4	50.00	<0.05	1-2-11-12 NO	Contiguous range expansion.
CP2	2-5	11.00	<0.05	1-2-3-4 NO	Restricted gene flow with isolation by distance.
CP1	2-6	8.00	<0.05	1-19-20-NO	Inadequate geographic sampling.
SP1	2-7	291.46	<0.05	1-2 IO	Inconclusive outcome.
HA + NP1 + NP2 + SP2	3-1	548.03	<0.05	1-2-11-12-13-14 NO	Long-distance colonization and/or past fragmentation.
CP1 + CP2 + SP1	3-2	271.93	<0.05	1-2-11-12 NO	Contiguous range expansion.
	Total cladogram	331.13	<0.05	1-2 IO	Inconclusive outcome.

Historical fragmentation followed by isolation by distance due to restricted gene flow was inferred for clade 1-4 (most haplotypes of NP2). Processes of restricted gene flow and/or long-distance dispersal in intermediate areas not occupied by the species, or gene flow followed by extinction of intermediate populations were inferred for clade 1-5 (most haplotypes of NP1). Clades 1-7 (most haplotypes of SP2) and 3-1 (HA, NP1, NP2, and SP2) have experienced long-distance colonization and/or fragmentation. Processes of restricted gene flow and/or long-distance dispersal were inferred for clade 1-9 (part of SP1). Isolation or long-distance dispersal was inferred for clade 1-10 (most haplotypes of SP1). Clades 2-2 (SP2) and 2-5 (CP2) have experienced restricted gene flow with isolation by distance. Finally, range expansion was inferred for clades 2-4 (NP2) and 3-2 (CP1, CP2, PS1, and PS2; Table 3, Fig. 3).

### Haplotype diversity

Considering all individuals from all 63 sampled populations, haplotype diversity (*h*) ranged from 0 to 0.821 with an average of 0.532, nucleotide diversity (*π*) ranged from 0 to 0.00218 with an average of 0.00128, and mean number of pairwise differences among haplotypes within populations (*p*) ranged from 0 and 3.857 with an average of 1.175 (Table 4).

**Table 4:** Diversity indices of sampled *Nassauvia* subgen. *Strongyloma* populations: haplotype (*h*) and nucleotide (*π*) diversity, and mean number of pairwise differences (*p*) with their respective standard deviation (DE)

*N*_loc_	*N*_hap_ (*N*_ind_)	*h* ± DE	*π* ± DE	*p* ± DE
1	H8 (7)	0.00000	0.00000	0.00000
2	H9 (8)	0.00000	0.00000	0.00000
3	H9 (7)	0.00000	0.00000	0.00000
4	H1 (7)	0.00000	0.00000	0.00000
5	H1 (1), H7 (2), H38 (2)	0.80000 ± 0.16400	0.00131 ± 0.00032	2.40000 ± 0.93915
6	H12 (6), H13 (2)	0.42900 ± 0.16900	0.00023 ± 0.00009	0.42900 ± 0.20248
7	H41 (2)	0.00000	0.00000	0.00000
8	H6 (3)	0.00000	0.00000	0.00000
9	H1 (5), H12 (1), H41 (2)	0.60700 ± 0.16400	0.00167 ± 0.00055	3.07100 ± 0.87407
10	H1 (6), H10 (1), H11 (1)	0.46400 ± 0.20000	0.00037 ± 0.00017	0.67900 ± 0.27203
11	H1 (8)	0.00000	0.00000	0.00000
12	H10 (8)	0.00000	0.00000	0.00000
13	H1 (7), H12 (1)	0.25000 ± 0.18000	0.00027 ± 0.00020	0.50000 ± 0.22361
14	H1 (2), H12 (4)	0.53300 ± 0.17200	0.00058 ± 0.00019	1.06700 ± 0.44271
15	H12 (2)	0.00000	0.00000	0.00000
16	H1 (6), H10 (2)	0.42900 ± 0.16900	0.00023 ± 0.00009	0.42900 ± 0.20248
17	H12 (1), H14 (4)	0.40000 ± 0.23700	0.00022 ± 0.00013	0.40000 ± 0.24900
18	H12 (3), H16 (1)	0.50000 ± 0.26500	0.00027 ± 0.00014	0.50000 ± 0.32711
19	H24 (1), H35 (4)	0.40000 ± 0.23700	0.00087 ± 0.00052	1.60000 ± 0.67305
20	H27 (1)	–	–	–
21	H27 (2), H28 (5)	0.47600 ± 0.17100	0.00052 ± 0.00019	0.95200 ± 0.37283
22	H27 (1), H30 (1)	–	–	–
23	H12 (2)	0.00000	0.00000	0.00000
24	H1 (1), H28 (1)	–	–	–
25	H24 (6)	0.00000	0.00000	0.00000
26	H25 (2)	0.00000	0.00000	0.00000
27	H1 (4)	0.00000	0.00000	0.00000
28	H15 (8)	0.00000	0.00000	0.00000
29	H17 (1)	–	–	–
30	H1 (4), H2 (1), H4 (2)	0.66700 ± 0.16000	0.00041 ± 0.00013	0.76200 ± 0.31780
31	H3 (2), H17 (2), H40 (4)	0.71400 ± 0.12300	0.00210 ± 0.00041	3.85700 ± 1.06583
32	H1 (8)	0.00000	0.00000	0.00000
33	H18 (1)	–	–	–
34	H5 (8)	0.00000	0.00000	0.00000
35	H18 (7)	0.00000	0.00000	0.00000
36	H25 (3), H33 (3)	0.60000 ± 0.12900	0.00033 ± 0.00007	0.60000 ± 0.29496
37	H25 (1), H26 (3), H34 (4)	0.67900 ± 0.12200	0.00123 ± 0.00019	2.25000 ± 0.67231
38	H25 (7)	0.00000	0.00000	0.00000
39	H25 (4), H31 (4)	0.57100 ± 0.09400	0.00031 ± 0.00005	0.57100 ± 0.24290
40	H39 (8)	0.00000	0.00000	0.00000
41	H25 (8)	0.00000	0.00000	0.00000
42	H1 (1), H18 (4), H19 (3)	0.67900 ± 0.12200	0.00043 ± 0.00011	0.78600 ± 0.30166
43	H25 (1)	–	–	–
44	H25 (2), H37 (3), H39 (3)	0.75000 ± 0.09600	0.00082 ± 0.00011	1.50000 ± 0.48580
45	H25 (8)	0.00000	0.00000	0.00000
46	H18 (4), H36 (2)	0.53300 ± 0.17200	0.00087 ± 0.00028	1.60000 ± 0.60332
47	H22 (1)	–	–	–
48	H18 (5), H25 (3)	0.53600 ± 0.12300	0.00117 ± 0.00027	2.14300 ± 0.64653
49	H23 (1), H25 (7)	0.25000 ± 0.18000	0.00041 ± 0.00029	0.75000 ± 0.29155
50	H25 (3), H32 (5)	0.53600 ± 0.12300	0.00029 ± 0.00007	0.53600 ± 0.23238
51	H18 (2), H20 (1)	0.66700 ± 0.31400	0.00036 ± 0.00017	0.66700 ± 0.47117
52	H25 (6)	0.00000	0.00000	0.00000
53	H25 (7), H30 (1)	0.25000 ± 0.18000	0.00014 ± 0.00010	0.25000 ± 0.14491
54	H18 (6)	0.00000	0.00000	0.00000
55	H25 (4)	0.00000	0.00000	0.00000
56	H18 (7), H20 (1)	0.25000 ± 0.18000	0.00014 ± 0.00010	0.25000 ± 0.14491
57	H18 (2), H20 (2), H21 (3), H25 (1)	0.82100 ± 0.10100	0.00142 ± 0.00042	2.60700 ± 0.76026
58	H25 (4), H29 (3)	0.57100 ± 0.11900	0.00031 ± 0.00007	0.57100 ± 0.26077
59	H25 (7)	0.00000	0.00000	0.00000
60	H25 (8)	0.00000	0.00000	0.00000
61	H25 (3)	0.00000	0.00000	0.00000
62	H25 (7)	0.00000	0.00000	0.00000
63	H25 (5)	0.00000	0.00000	0.00000

With one exception, the highest values of *h* were found in populations of central-western Patagonia (populations 5, 9, 30, 31, 36, 37, 42, 44, and 51; Table 1, Fig. 1), located from the flanks of the Andes up to 69°W, and latitudinally from 40°S in southern Neuquén to 50°S in southern Santa Cruz. Population 57 had the highest haplotype diversity and occurs outside this range in south-eastern Santa Cruz. The highest values of *π* also occur in populations of central-western Patagonia, coinciding with most populations that showed higher haplotype diversity (populations 5, 9, 31, 37, 48, and 57), and in one population (24) located in south-eastern Santa Cruz. The highest values of *p* similarly occur in populations of central-western Patagonia, coinciding with most populations that showed higher haplotype and nucleotide diversity (populations 5, 9, 31, 37, 48, and 57). Most populations with high genetic diversity also had exclusive haplotypes (populations 5, 30, 31, 36, 37, 42, 44, and 57, Table 4).

The same diversity indices were calculated for each geographic region (Table 5, Fig. 3). The NP2 region showed the highest value of haplotype diversity (*h* = 0.800), followed by the CP2 region, which also showed the highest values of nucleotide diversity and pairwise differences (*h* = 0.727; *π* = 0.00084; *p* = 1.527), while the NP1 region showed the lowest values of *h*, *π*, and *p* (*h* = 0.399; *π* = 0.00027; *p* = 0.488).

**Table 5:** Diversity indices and results of demographic analyses used to test range expansion in *Nassauvia* subgen. *Strongyloma* for each geographic area in Argentina. Haplotype (*h*) and nucleotide (*π*) diversity, mean number of pairwise differences (*p*), Tajima's *D*, Fu's *F*_S_, and Ramos-Onsins and Rozas's *R*_2_ are shown

	Diversity indices			Demographic analyses		
Geographic region	*h* ± SD	*π* ± SD	*p* ± SD	*D*	*F*_S_	R_2_
High-Andean	0.45500 ± 0.07800	0.00049 ± 0.00008	0.90900 ± 0.19493	1.48848	2.78400	0.22730
Northern Patagonia 1	0.39900 ± 0.06800	0.00027 ± 0.00005	0.48800 ± 0.07071	−1.40325	−4.18000*	0.04070*
Northern Patagonia 2	0.80000 ± 0.03400	0.00064 ± 0.00006	1.16800 ± 0.16124	−0.73108	−2.54900	0.08250
Central Patagonia 1	0.57100 ± 0.09400	0.00124 ± 0.00021	2.28600 ± 0.68117	2.10118	3.93300	0.28570
Central Patagonia 2	0.72700 ± 0.06800	0.00084 ± 0.00011	1.52700 ± 0.41231	1.68091	1.49100	0.25450
Southern Patagonia 1	0.54600 ± 0.04800	0.00050 ± 0.00006	0.91600 ± 0.07071	−1.54473*	−7.32400*	0.03530*
Southern Patagonia 2	0.46300 ± 0.07930	0.00041 ± 0.00011	0.75500 ± 0.11832	−1.18369	−2.04400	0.06530
Total	0.88100 ± 0.01100	0.00178 ± 0.00005	3.24600 ± 0.12247	−1.38638	−20.51400	0.03850

Results consistent with demographic expansion are shown in bold.

* *P* < 0.05.

### Population genetic structure

Analyses of molecular variance (AMOVA; Table 6) showed significant differences among all sources of variation tested (*P* < 0.001), with the exception of regions located inside and outside the boundary of the GPG (Fig. 1). The AMOVA revealed that genetic variation of subgen. *Strongyloma* was best explained by differences among populations within regions that were glaciated or nonglaciated during GPG (73.6%, *P* < 0.001). By dividing the regions according to major rivers (Fig. 1), the highest percentage of variation was explained by differences among populations within each of the regions (50.6%, *P* < 0.001).

**Table 6:** Results of the analyses of molecular variance (AMOVA) for 63 populations of *Nassauvia* subgen. *Strongyloma* based on cpDNA sequence data. Degrees of freedom (df), sum of squared deviations (SSD), variance components (VC), percentage of total variance (% total), and significance value (*P*) are given for each hierarchical level

Source of variation	df	SSD	VC	% total	*P*
1. Regions divided by rivers					
Among regions	4	168.349	0.507	23.87	<0.001
Among populations within regions	44	356.604	1.075	50.60	<0.001
Within populations	297	161.099	0.542	25.53	<0.001
Total	345	686.052	2.125		
2. Glaciated versus nonglaciated regions during Great Patagonian Glaciation					
Among regions	1	10.703	−0.013	−0.68	n.s.
Among populations within regions	47	514.250	1.477	73.64	<0.001
Within populations	297	161.099	0.542	27.04	<0.001
Total	345	686.052	2.006		

n.s., not significant, *P* > 0.05.

### Past demographic analyses

Recent demographic expansion was evidenced for the NP1 and the SP1 regions. For the NP1 region, Fu's *F*_S_ and Ramos-Onsins and Rozas' *R*_2_ statistics showed a negative value of −4.18 and a small positive value of 0.0407**,** respectively. For the SP1 region, Tajima's *D* and Fu's *F*_S_ showed negative values of −1.54473 and −7.324, respectively, while Ramos-Onsins and Rozas's *R*_2_ showed a small positive value of 0.0353 (Table 5).

### Potential Pleistocene geographic distribution

**Figure 4:**
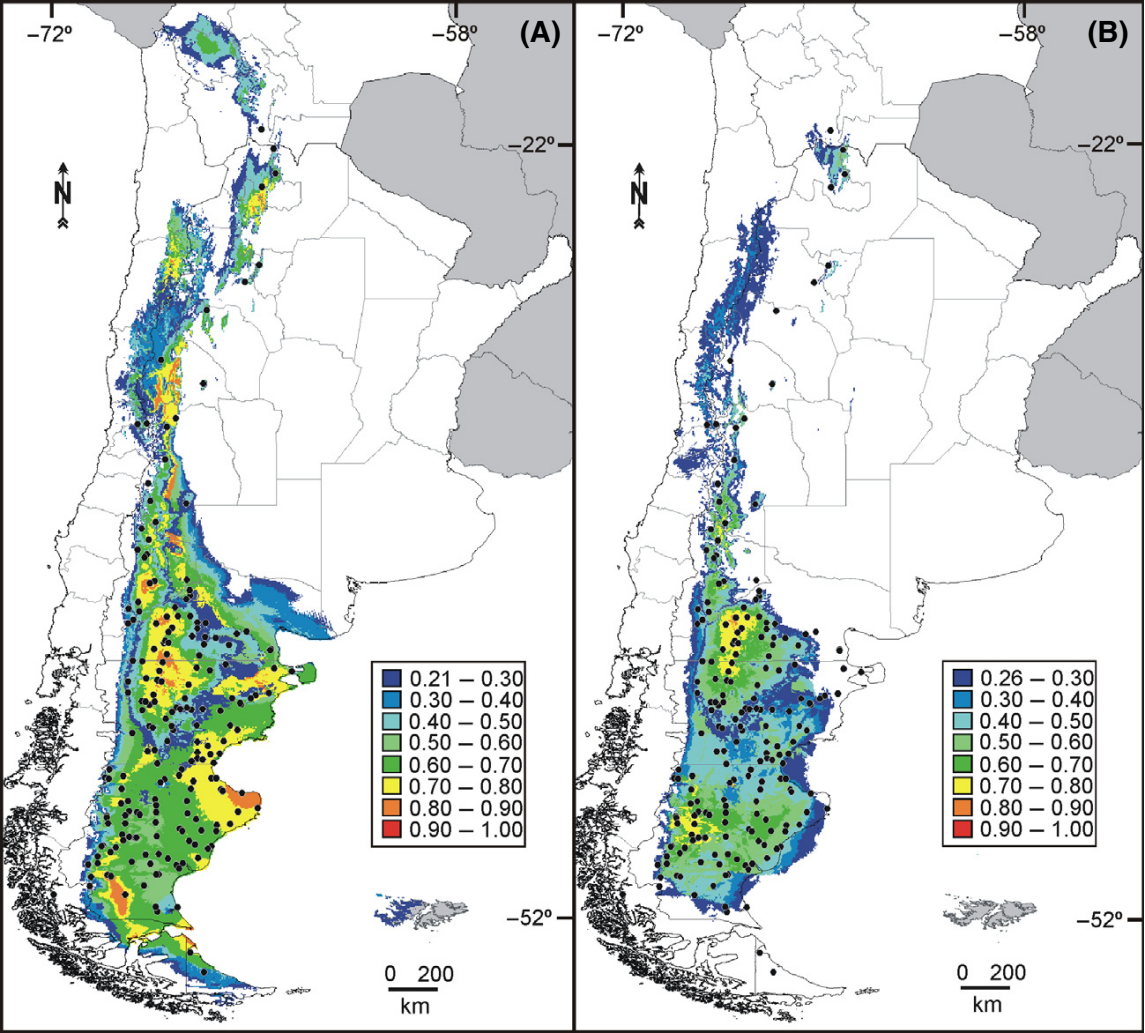
Predictive distribution models of *Nassauvia* subgen. *Strongyloma* for (A) the present, and (B) the last glacial maximum, showing habitat suitability in southern South America calculated with MaxEnt. Black dots refer to point localities on which the models are based.

Present and LGM species distribution models for subgen. *Strongyloma* are presented in Figure 4. In predicting the current distribution, the mean value of the AUC for the test data of the model for the present was 0.932 ± 0.008. Because the mean value of the AUC for the test data using the MIROC palaeomodel was 0.836 ± 0.075, compared with 0.789 ± 0.021 using the CCSM palaeomodel, the former was selected for projecting the distribution during the LGM.

The predicted model for the present (Fig. 4A) was a fairly good representation of the known geographic distribution for subgen. *Strongyloma*, although some inconsistencies between projected and realized distribution areas were found. Low probability (0.21–0.30) of suitable conditions was found in the Islas Malvinas/Falkland Islands, where these plants have never been observed.

The model of the geographic distribution for the LGM showed some important differences with the known current geographic distribution of the subgenus (Fig. 4B). Low (0.26–0.5) to null probability of suitable conditions was found in an area between the Chubut and the Deseado rivers and also in southern Santa Cruz and Tierra del Fuego. The prediction was overprojected over the current Atlantic continental shelf, extending approximately between 45°S (San Jorge Gulf, Chubut) and 51°S (Bahía Grande, Santa Cruz).

For the training data of the palaeomodel, the highest contribution and the highest permutation importance was for the annual mean temperature (Bio1 = 42.8% and 36.1%, respectively), indicating that this environmental variable contributed most to the construction of the model. In the jackknife test for the training data, the mean temperature of the coldest quarter (Bio11) presented the most useful information when used in isolation to build the model, causing the model to reach the highest gain and allowing a reasonable adjustment to the training data. The mean temperature of the wettest quarter (Bio8) contained a substantial amount of useful information that was not contained in the model when only the remaining variables were used, since excluding Bio8 in the model decreased training gain. Jackknife tests for the gain and for the AUC of the test data yielded similar results to the jackknife test for the gain of the training data, suggesting that mean winter temperature (i.e., the coldest and the wettest quarter) helped MaxEnt achieve a good fit to the training data and also to generalize better, achieving comparatively better results on the test data.

## Discussion

The phylogeographical and palaeomodelling analyses suggest that the genetic diversity and structure found in *Nassauvia* subgen. *Strongyloma* were shaped mainly by three main different historical events: (1) isolation in refugia and restricted gene flow during Pleistocene glacial periods, followed by postglacial range expansions; (2) colonization of new available areas during Pleistocene glaciations and postglacial retraction of populations; and (3) fragmentation of populations in areas not reached by the ice sheet during Pleistocene glacial periods followed by postglacial range expansions.

### Pleistocene refugia in central-western and south-eastern Patagonia

Our data suggest that populations of *Nassauvia* subgen. *Strongyloma* may have withstood the extreme cold and aridity during the Pleistocene glaciations in two kinds of refugia (Holderegger and Thiel-Egenter 2009): (1) peripheral glacial refugia—at least three along the eastern foothills of the Andes— located in south-western Neuquén (*c*. 39°30′ S 70°30′ W), north-western Chubut (*c*. 43°S 71°W), and north-western Santa Cruz (*c*. 47°S 71°W); and (2) lowland glacial refugia—at least four— located near the Chubut River in central-western Chubut (*c*. 44°S 69°30′ W), near the Deseado River in central-northern Santa Cruz (*c*. 46°30′ S 69°30′ W), and near the Chico River in central-western (*c*. 48°S 71°W), and south-eastern (*c*. 50°S 69°W) Santa Cruz.

The high diversity and several exclusive haplotypes found in these populations of subgen. *Strongyloma* are consistent with long-term habitation such as would be expected from Pleistocene refugia (Hewitt 1996; Taberlet and Cheddadi 2002; Petit et al. 2003; Provan and Bennett 2008). The finding of exclusive haplotypes suggests there would have been restricted gene flow and that isolation over time allowed the accumulation of genetic differences among populations. Most of these populations were found within the limits of the ice sheet of the GPG. Moreover, restricted gene flow and posterior range expansion were inferred by NCA and corroborated in most cases by demographic analyses, indicating that they might have survived in refugia during glacial periods, as suggested for other Patagonian organisms in the areas mentioned above (Sérsic et al. 2011; Sede et al. 2012; Cosacov et al. 2013). The highest probabilities of occurrence projected especially for central-western and south-eastern Patagonia, both for the current distribution, as well for the palaeodistribution, reinforce the conclusion that subgen. *Strongyloma* probably persisted in refugia in these areas, without drastic climatic restrictions.

### Colonization of eastern Patagonia during Pleistocene glaciations

During Pleistocene glaciations, the current Atlantic submarine platform was partially exposed as a result of the sea level lowering (*c*. 100–140 m during full glacial episodes), allowing for plant colonization (Rabassa 2008). Palaeomodelling supports that populations of *Nassauvia* subgen. *Strongyloma* could have inhabited that exposed area of the coast. After glaciations, and as the result of the sea level rise, populations were forced to retract to the current Atlantic coast. Low diversity and the absence of exclusive haplotypes in eastern Patagonia are then explained by the expansion of populations over the current Atlantic coast during Pleistocene glaciations, and their recent, postglacial retraction, with the consequent loss of genetic diversity.

### Population fragmentation in central Patagonia and phylogeographical breaks

Latitudinal discontinuities have been previously reported for other Patagonian organisms, both plants and animals (Sérsic et al. 2011; Sede et al. 2012; Cosacov et al. 2013). Dynamics of the river basins, palaeobasins, and coastline shift during the Pleistocene glacial–interglacial cycles constitute factors that may have converted Patagonia into a highly fragmented landscape (Sérsic et al. 2011). In particular, the Chubut and the Deseado river basins may have been important historical barriers that facilitated isolation between populations of different Patagonian species that persisted north and/or south of these river basins (e.g., *Phyllotis xanthopygus*, Kim et al. 1998; *Hordeum* spp., Jakob et al. 2009; *Mulinum spinosum*, Sede et al. 2012; *Anarthrophyllum desideratum*, Cosacov et al. 2013). However, adverse climatic conditions such as lower winter temperature and precipitation during glacial periods have been suggested as a possible reason for plant population fragmentation in the same geographic area (Sede et al. 2012; Cosacov et al. 2013). Our palaeomodelling results also support a low probability of suitable climatic conditions for *Nassauvia* subgen. *Strongyloma* during the LGM in the center of the steppe, between the Chubut (*c*. 44°S) and the Deseado (*c*. 47°S) rivers. In fact, the geographic distribution of this subgenus, as in the steppe shrub *M. spinosum* (Sede et al. 2012), seems to be influenced mainly by the mean temperature of the winter, a biologically important variable that defines the start of the growing season (Paruelo et al. 1998). Our findings also support the traditional water balance hypothesis, which considers aridization as the main factor affecting plant population demography and diversification in Patagonia (Cosacov et al. 2013), related to the enlargement and reduction of the main river basins.

Based on past river basin dynamics and climate conditions cited above, a significant difference could be expected between northern and southern haplotypes within subgen. *Strongyloma*. Nevertheless, there is a close genetic relation between a few northern and central-northern lineages with southern lineages distributed in distant areas. However, a general northern–southern haploclade structure can be supported combining the above environmental evidence with NCA inferences and demographic analyses, in two historical steps: (1) both fragmentation between northern and southern haploclades, and isolation with restricted gene flow of one southern haploclade during Pleistocene glaciations, produced the split of the original distribution into two groups of population, one north of the Chubut river basin, and one south of the Deseado river basin; and (2) successive range expansions after the Pleistocene glaciations, mainly toward warmer and wetter conditions in the center of the Patagonian steppe. The most likely posterior demographic expansions would be from both northern and southern populations toward central-eastern Chubut Province. The expansion of northern, central, and southern haploclades could have generated recent contact areas throughout the entire Patagonian steppe, but mainly between the Chubut and the Deseado rivers.

Within a general latitudinal geographic structure of haploclades, other phylogeographical breaks can be distinguished related to river basins. On one hand, the high-Andean haploclade located just north of the Agrio and Neuquén rivers at *c*. 38°S was clearly separated from the rest, in accordance with a latitudinal break reported for a lizard species (*Liolaemus elongatus*, Morando et al. 2003). Low diversity and the presence of only one exclusive haplotype in high-Andean populations may be explained by recent colonization or this was a poor area for the survival of subgen. *Strongyloma* during the LGM. However, these results should be interpreted with caution because of incomplete sampling of high-Andean region. On the other hand, the northern limit of one central haploclade (CP1) coincides with the break at Limay River basin reported for another lizard species (*Liolaemus bibronii*, Morando et al. 2007).

Finally, according to haplotype analyses, the four most widespread haplotypes and several other interior and tip haplotypes were shared among populations of different morphological variants, suggesting that there is no strict relation between morphology and genetic diversity. Furthermore, throughout the entire Patagonian steppe, some populations have haplotypes from different clades. This indication of distantly related haplotypes co-occurring suggests the existence of numerous contact zones (Avise 2000) throughout the steppe: within northern lineages in central Río Negro; between northern and central clades in central-western Río Negro; between northern and southern lineages in southern Neuquén, north-western Chubut, and north-western Santa Cruz; between central and southern clades in central-northern Santa Cruz; and within southern lineages in central, south-western, and south-eastern Santa Cruz.

## Conclusion

Phylogeographical and palaeomodelling patterns found in *Nassauvia* subgen. *Strongyloma* appear to reflect climatic fluctuations and landscape changes that occurred during the Pleistocene having an impact on the distribution, demography, and diversification of different lineages within the group. Past events differentially affected areas within the immense geographic range of these shrubs. During glaciations, subgen. *Strongyloma* persisted in peripheral refugia along the eastern foothills of the Andes, in lowland refugia in central-western and south-eastern Patagonia, over the exposed Atlantic submarine platform toward the east, and in fragmented populations north and south the Chubut and the Deseado river basins. Related to those past scenarios, Pleistocene glaciations indirectly affected the demography of this group of plants through decreased winter temperatures and water availability in different areas of its immense geographic range. Future comparative phylogeographical studies should consider patterns found in subgen. *Strongyloma* to better comprehend the processes that have affected the Patagonian biota.

## References

[b1] Acosta MC, Premoli AC (2010). Evidence of chloroplast capture in South American *Nothofagus* (subgenus *Nothofagus*, Nothofagaceae). Mol. Phylogenet. Evol.

[b2] Amico GC, Nickrent DL (2009). Population structure and phylogeography of the mistletoes *Tristerix corymbosus* and *T. aphyllus* (Loranthaceae) using chloroplast DNA sequence variation. Am. J. Bot.

[b3] Avise JC (2000). Phylogeography: the history and formation of species.

[b4] Barreda V, Palazzesi L (2007). Patagonian vegetation turnovers during the Paleogene-Early Neogene: origin of arid-adapted floras. Bot. Rev.

[b5] Barreda V, Palazzesi L, Tellería MC (2008). Fossil pollen grains of Asteraceae from the Miocene of Patagonia: Nassauviinae affinity. Rev. Palaeobot. Palynol.

[b6] Beaumont MA, Panchal M (2008). On the validity of nested clade phylogeographical analysis. Mol. Ecol.

[b7] Cabrera AL (1982). Revisión del género *Nassauvia* (Compositae). Darwiniana.

[b8] Cabrera AL, Willink W (1980). Biogeografía de América Latina. Serie de Biología, Monografía 13. Ed. 2, corr. Colección de Monografías Científicas de la Secretaría General de la Organización de los Estados Americanos.

[b9] Clement M, Posada D, Crandall KA (2000). TCS: a computer program to estimate gene genealogies. Mol. Ecol.

[b10] Correa MN (1998). Flora Patagónica, Parte I. Colección Científica del INTA.

[b11] Cosacov A, Sérsic AN, Sosa V, Johnson LA, Cocucci A (2010). Multiple periglacial refugia in the Patagonian steppe and post-glacial colonization of the Andes: the phylogeography of *Calceolaria polyrhiza*. J. Biogeogr.

[b12] Cosacov A, Johnson LA, Paiaro V, Cocucci AA, Córdoba FE, Sérsic AN (2013). Precipitation rather than temperature influenced the phylogeography of the endemic shrub *Anarthrophyllum desideratum* in the Patagonian steppe. J. Biogeogr.

[b13] Crandall KA, Templeton AR (1993). Empirical tests of some predictions from coalescent theory with applications to intraspecific phylogeny reconstruction. Genetics.

[b14] Cullings KW (1992). Design and testing of a plant specific PCR primer for ecological and evolutionary studies. Mol. Ecol.

[b15] Di Rienzo JA, Casanoves F, Balzarini MG, González L, Tablada M, Robledo CW (2011). InfoStat version 2011. Grupo InfoStat.

[b16] Doyle JJ, Doyle JL (1987). A rapid DNA isolation procedure from small quantities of fresh leaf tissues. Phytochem. Bull.

[b17] Excoffier L, Lischer HEL (2010). Arlequin suite version 3.5: a new series of programs to perform population genetics analyses under Linux and Windows. Mol. Ecol. Resour.

[b18] Freire SE, Crisci JV, Katinas L (1993). A cladistic analysis of *Nassauvia* Comm. ex Juss. (Asteraceae, Mutisieae) and related genera. Bot. J. Linn. Soc.

[b19] Fu YX (1997). Statistical tests of neutrality of mutations against population growth, hitchhiking and background selection. Genetics.

[b20] Funk VA, Susanna A, Stuessy TF, Bayer RJ (2009). Systematics, evolution, and biogeography of Compositae.

[b21] Hall TA (1999). BioEdit: a user-friendly biological sequence alignment editor and analysis program for Windows 95/98/NT. Nucleic Acids Symp. Ser.

[b22] Hewitt GM (1996). Some genetic consequences of ice ages, and their role in divergence and speciation. Biol. J. Linn. Soc.

[b23] Hijmans RJ, Graham CH (2006). The ability of climate envelope models to predict the effect of climate change on species distributions. Glob. Chang. Biol.

[b24] Hijmans RJ, Guarino L, Cruz M, Rojas E (2001). Computer tools for spatial analysis of plant genetic resources data: 1. DIVA-GIS. Plant Genet. Resour. Newsl.

[b25] Hijmans RJ, Cameron SE, Parra JL, Jones PG, Jarvis A (2005). Very high resolution interpolated climate surfaces for global land areas. Int. J. Climatol.

[b26] Holderegger R, Thiel-Egenter C (2009). A discussion of different types of glacial refugia used in mountain biogeography and phylogeography. J. Biogeogr.

[b27] Jakob SS, Martinez-Meyer E, Blattner FR (2009). Phylogeographic analyses and paleodistribution modeling indicate Pleistocene *in situ* survival of *Hordeum* species (Poaceae) in southern Patagonia without genetic or spatial restriction. Mol. Biol. Evol.

[b28] Kim I, Phillips CJ, Monjeau JA, Birney EC, Noack K, Pumo DE (1998). Habitat islands, genetic diversity, and gene flow in a Patagonian rodent. Mol. Ecol.

[b29] Knowles LL (2008). Why does a method that fails continue to be used?. Evolution.

[b30] Librado P, Rozas J (2009). DnaSP version 5: a software for comprehensive analysis of DNA polymorphism data. Bioinformatics.

[b31] Maraner F, Samuel R, Stuessy TF, Crawford DJ, Crisci JV, Pandey A (2012). Molecular phylogeny of *Nassauvia* (Asteraceae, Mutisieae) based on nrDNA ITS sequences. Plant Syst. Evol.

[b32] Marchelli P, Gallo LA (2006). Multiple ice-age refugia in a southern beech of South America as evidenced by chloroplast DNA markers. Conserv. Genet.

[b33] Markgraf V, McGlone M, Hope G (1995). Neogene paleoenvironmental and paleoclimatic change in southern temperate ecosystems – a southern perspective. Trends Ecol. Evol.

[b34] Morando M, Ávila LJ, Sites JW (2003). Sampling strategies for delimiting species: genes, individuals, and populations in the *Liolaemus elongatus-kriegi* complex (Squamata: Liolaemidae) in Andean-Patagonian South America. Syst. Biol.

[b35] Morando M, Ávila LJ, Turner CR, Sites JW (2007). Molecular evidence for a species complex in the patagonian lizard *Liolaemus bibronii* and phylogeography of the closely related *Liolaemus gracilis* (Squamata: Liolaemini). Mol. Phylogenet. Evol.

[b36] Muellner AN, Tremetsberger K, Stuessy T, Baeza CM (2005). Pleistocene refugia and recolonization routes in the southern Andes: insights from *Hypochaeris palustris* (Astraceae, Lactuceae). Mol. Ecol.

[b37] Müller K (2005). SeqState: primer design and sequence statistics for phylogenetic DNA data sets. Appl. Bioinformatics.

[b38] Nei M (1987). Molecular evolutionary genetics.

[b39] Nicola MV, Johnson LA, Pozner R (2014). Geographic variation amongst closely related, highly variable species with a wide distribution range: the South Andean-Patagonian *Nassauvia* subgenus *Strongyloma* (Asteraceae, Nassauvieae). Syst. Bot.

[b40] Ogg JG, Ogg GM, Gradstein FM (2008). The Concise Geologic Time scale.

[b41] Panchal M (2007). The automation of nested clade phylogeographic analysis. Bioinformatics.

[b42] Paruelo JM, Beltrán A, Jobbágy E, Sala OE, Golluscio RA (1998). The climate of Patagonia: general patterns and controls on biotic processes. Ecol. Austral.

[b43] Peterson AT, Nakazawa Y (2008). Environmental data sets matter in ecological niche modelling: an example with *Solenopsis invicta* and *Solenopsis richteri*. Global Ecol. Biogeogr.

[b44] Petit RJ (2007). The coup de grâce for the nested clade phylogeographic analysis?. Mol. Ecol.

[b45] Petit RJ, Aguinagalde I, de Beaulieu JL, Bittkau C, Brewer S, Cheddadi R (2003). Glacial refugia: hotspots but not melting pots of genetic diversity. Science.

[b46] Phillips SJ, Dudík M, Brodley CE, Schapire RE (2004). A maximum entropy approach to species distribution modeling. Proceedings of the Twenty-First International Conference on Machine Learning.

[b47] Posada D, Crandall KA, Templeton AR (2000). GeoDis: a program for the cladistic nested analysis of the geographical distribution of genetic haplotypes. Mol. Ecol.

[b48] Provan J, Bennett KD (2008). Phylogeographic insights into cryptic glacial refugia. Trends Ecol. Evol.

[b49] Rabassa J, Rabassa J (2008). Late Cenozoic Glaciations in Patagonia and Tierra del Fuego. The late Cenozoic of Patagonia and Tierra del Fuego.

[b50] Rabassa J, Coronato A, Martínez O (2011). Late Cenozoic glaciations in Patagonia and Tierra del Fuego: an updated review. Biol. J. Linn. Soc.

[b51] Ramos-Onsins SE, Rozas J (2002). Statistical properties of new neutrality tests against population growth. Mol. Biol. Evol.

[b52] Sede SM, Nicola MV, Pozner R, Johnson LA (2012). Phylogeography and palaeodistribution modelling in the Patagonian steppe: the case of *Mulinum spinosum* (Apiaceae). J. Biogeogr.

[b53] Segovia RA, Pérez MF, Hinojosa LF (2012). Genetic evidence for glacial refugia of the temperate tree *Eucryphia cordifolia* (Cunoniaceae) in southern South America. Am. J. Bot.

[b54] Sérsic AN, Cosacov A, Cocucci AA, Johnson LA, Pozner R, Ávila LJ (2011). Emerging phylogeographic patterns of plants and terrestrial vertebrates from Patagonia. Bot. J. Linn. Soc.

[b55] Shaw J, Lickey EB, Schilling EE, Small RL (2007). Comparison of whole chloroplast genome sequences to choose noncoding regions for phylogenetic studies in angiosperms: the tortoise and the hare III. Am. J. Bot.

[b56] Simmons MP, Ochoterena H (2000). Gaps as characters in sequence-based phylogenetic analyses. Syst. Biol.

[b57] Taberlet P, Cheddadi R (2002). Quaternary refugia and persistence of biodiversity. Science.

[b58] Tajima F (1983). Evolutionary relationship of DNA sequences in finite populations. Genetics.

[b59] Tajima F (1989). The effect of change in population size on DNA polymorphism. Genetics.

[b60] Templeton AR (2004). Statistical phylogeography: methods of evaluating and minimizing inference errors. Mol. Ecol.

[b61] Templeton AR (2008). Nested clade analysis: an extensively validated method for strong phylogeographic inference. Mol. Ecol.

[b62] Templeton AR (2009). Why does a method that fails continue to be used? The answer. Evolution.

[b63] Templeton AR (2011). http://darwin.uvigo.es/download/geodisKey_06Jan11.pdf.

[b64] Templeton AR, Sing CF (1993). A cladistic analysis of phenotypic associations with haplotypes inferred from restriction endonuclease mapping. IV. Nested analyses with cladogram uncertainty and recombination. Genetics.

[b65] Templeton AR, Boerwinkle E, Sing CF (1987). A cladistic analysis of phenotypic associations with haplotypes inferred from restriction endonuclease mapping.1. Basic theory and an analysis of alcohol dehydrogenase activity in drosophila. Genetics.

[b66] Templeton AR, Routman E, Phillips CA (1995). Separating population structure from population history: a cladistic analysis of the geographical distribution of mitochondrial DNA haplotypes in the tiger salamander, *Ambystoma tigrinum*. Genetics.

[b67] Thompson JD, Gibson TJ, Plewniak F, Jeanmougin F, Higgins DG (1997). The CLUSTAL_X windows interface: flexible strategies for multiple sequence alignment aided by quality analysis tools. Nucleic Acids Res.

[b68] Vidal-Russell R, Souto CP, Premoli AC (2011). Multiple Pleistocene refugia in the widespread Patagonian tree *Embothrium coccineum* (Proteaceae). Aust. J. Bot.

[b69] Werneck FP, Costa GC, Colli GR, Prado DE, Sites JW (2011). Revisiting the historical distribution of seasonally dry tropical forests: new insights based on palaeodistribution modelling and palynological evidence. Global Ecol. Biogeogr.

